# Diverse functions of NLRP3 inflammasome in PANoptosis and diseases

**DOI:** 10.1038/s41420-025-02689-1

**Published:** 2025-08-19

**Authors:** Yuanyuan Jiang, Zhiyuan Qiang, Yue Liu, Liwei Zhu, Long Xiao, Zhenfang Du, Rong Cai, Sheng Qiang

**Affiliations:** 1 Translational Medical Innovation Center, Zhangjiagang TCM Hospital Affiliated to Nanjing University of Chinese Medicine, Zhangjiagang, Jiangsu China; 2 School of Medicine Southeast University, Nanjing, Jiangsu China

**Keywords:** Cell death and immune response, Inflammasome

## Abstract

NOD-like receptor (NLR) family pyrin domain-containing 3 (NLRP3) is a vital sensor of the innate immune system, capable of responding to various exogenous invading pathogens and endogenous cell injury. Once the danger signal is detected, NLRP3 recruits downstream molecules to assemble into inflammasomes, which induce inflammatory cell death and trigger an inflammatory response. PANoptosis is a specific mode of inflammatory cell death that integrates the processes of pyroptosis, apoptosis, and necrosis. It is primarily driven by a multiprotein complex termed the PANoptosome. The NLRP3 inflammasome, an essential component of the PANoptosome, is implicated in the pathogenesis of several human disorders. Targeted inhibition of NLRP3 activation specifically has a notable impact on mitigating a variety of disease conditions. This review briefly describes how the NLRP3 inflammasome forms and is activated, outlines its multifaceted roles in disorders, and emphasizes the vital role of NLRP3 in PANoptosis. Additionally, we discuss the potential of NLRP3 as a target for the clinical management of associated diseases. Improved understanding of the NLRP3 inflammasome and its involvement in PANoptosis is crucial for guiding new treatment strategies.

## Facts

- The NLRP3 inflammasome participates in numerous inflammatory cell deaths.
- NLRP3 has an essential function in mediating PANoptosis, a unique form of cell death identified in recent years.
- NLRP3 inhibitors are highly effective in various human diseases.

## Open Questions

- How exactly is the NLRP3 inflammasome activated at the molecular level?
- How is the NLRP3 inflammasome involved in mediating PANoptosis?
- How can safe and effective NLRP3 inhibitors be developed for treating diseases in humans?

## Introduction

The innate immune system can initiate a swift immune response through different pattern recognition receptors (PRRs), which serve as the primary defense against pathogen invasion and homeostatic perturbations [1, 2]. NLRP3-mediated pathogenic mechanisms were first identified in cryopyrin-associated periodic syndrome (CAPS), a hereditary autoinflammatory condition transmitted through dominant inheritance patterns [3]. These patients with *NLRP3* gain-of-function mutations usually present with clinical features of periodic fevers, arthritis, and urticaria [4]. Earlier studies focused on the link between NLRP3 and IL-1-mediated inflammation and revealed that NLRP3 contributes to the progression of autoinflammatory diseases [5]. In parallel, it has been discovered that NLRP3, as a sensor, recruits the adapter protein ASC (apoptosis-associated speck-like protein containing a C-terminal caspase-recruitment domain) through the interaction of homologous amino-terminal pyrin domains (PYDs) after encountering various stimuli, including pathogen-associated molecular patterns (PAMPs) and damage-associated molecular patterns (DAMPs). The ASC speck functions as a platform for the recruitment of pro-caspase-1 through CARD-CARD domain binding, completing the formation of the NLRP3 inflammasome, which triggers caspase-1 to self-cleave and activate [6]. The activated caspase-1 subsequently cleaves gasdermin D (GSDMD), generating N-terminal fragments that are inserted into the cell membrane, where they form channels for IL-1β and IL-18 cytokine release [7]. Not only is the NLRP3 inflammasome essential for mediating host innate immune defense mechanisms and maintaining homeostasis, but it can also contribute to immunopathology in some diseases such as CAPS, atherosclerosis, multiple sclerosis (MS), and cancers [8]. Therefore, NLRP3 is recognized as a next-generation candidate target for numerous immune disorders due to its less immunosuppressive impact compared to existing anti-IL-1 treatments.

The process of cell death is acknowledged to be irreversible and serves both physiological and pathological functions. Generally, it leads to the termination of corresponding functions and can result in bodily impairment. Nevertheless, several physiological processes, including embryonic development and immune selection of B cells, require cell death to remove damaged or obsolete cells. Additionally, this process reduces the harmful spread by eliminating pathogen-infected cells [9]. The cell death pathway can be classified into non-lytic (largely immunologically silent) and lytic (pro-inflammatory) types. Apoptosis, a non-lytic form, is distinguished by the generation of apoptotic bodies, cytoplasmic vacuolation, nuclear condensation, and cytoskeletal degradation [10]. By contrast, some cell death occurs through the lytic pathway, which involves cell bursting and the secretion of cytoplasmic pro-inflammatory contents. Pyroptosis and necroptosis are currently two of the most widely and deeply studied lytic pathways [9]. For a long period, apoptosis has been known as a highly coordinated and immunologically “silent” process of cell death. In contrast, pyroptosis and necroptosis are regarded as “alarms” that release pro-inflammatory signals, triggering an inflammatory response [11]. These forms of cell death are collectively known as programmed cell death (PCD), which involves tightly regulated and complex molecular effector mechanisms [12]. Recently, a new cell death pattern known as PANoptosis has attracted significant interest from researchers. It is described as a lytic and inflammatory PCD pathway governed by PANoptosome complexes, which combine the essential characteristics of pyroptosis, apoptosis, and necrosis; however, it does not fit neatly into any of these categories [13]. PANoptosis, a distinct type of inflammatory cell death, is reported to have a key function in various diseases, such as infections, inflammatory diseases, and tumors [14]. Recent studies reveal that NLRP3 is an important element in the assembly of the PANoptosome complex, which leads to PANoptosis [15]. Therefore, NLRP3 inflammasome-mediated excessive cell death may cause adverse inflammation and could be targeted for treating various diseases.

This review provides an overview of the mechanisms that initiate and activate the NLRP3 inflammasome, as well as its inseparable relationship with PANoptosis. It summarizes the latest advancements in potential NLRP3 inhibitors, offering attractive prospects for treatment strategies of related diseases.

## NLRP3 inflammasome activation

Inflammasomes, which are polymeric cytoplasmic protein complexes, trigger inflammatory responses and generally consist of a sensor protein, the adapter ASC, and the effector pro-caspase-1. Many sensor proteins of inflammasomes need to be activated by specific pathogen signals, such as NLRC4, which is primarily stimulated by bacterial flagella or the Salmonella type III secretion system (T3SS) [16]. Uniquely, the NLRP3 inflammasome exhibits broad activation specificity, responding to diverse PAMPs and DAMPs, including viral RNAs, pore-forming toxins, extracellular ATP, and particulate matter [17]. Current research classifies the mechanisms by which the NLRP3 inflammasome is activated into three distinct pathways: canonical, non-canonical, and one-step activation (also referred to as alternative activation).

### Canonical activation

#### The priming step

**Fig. 1:**
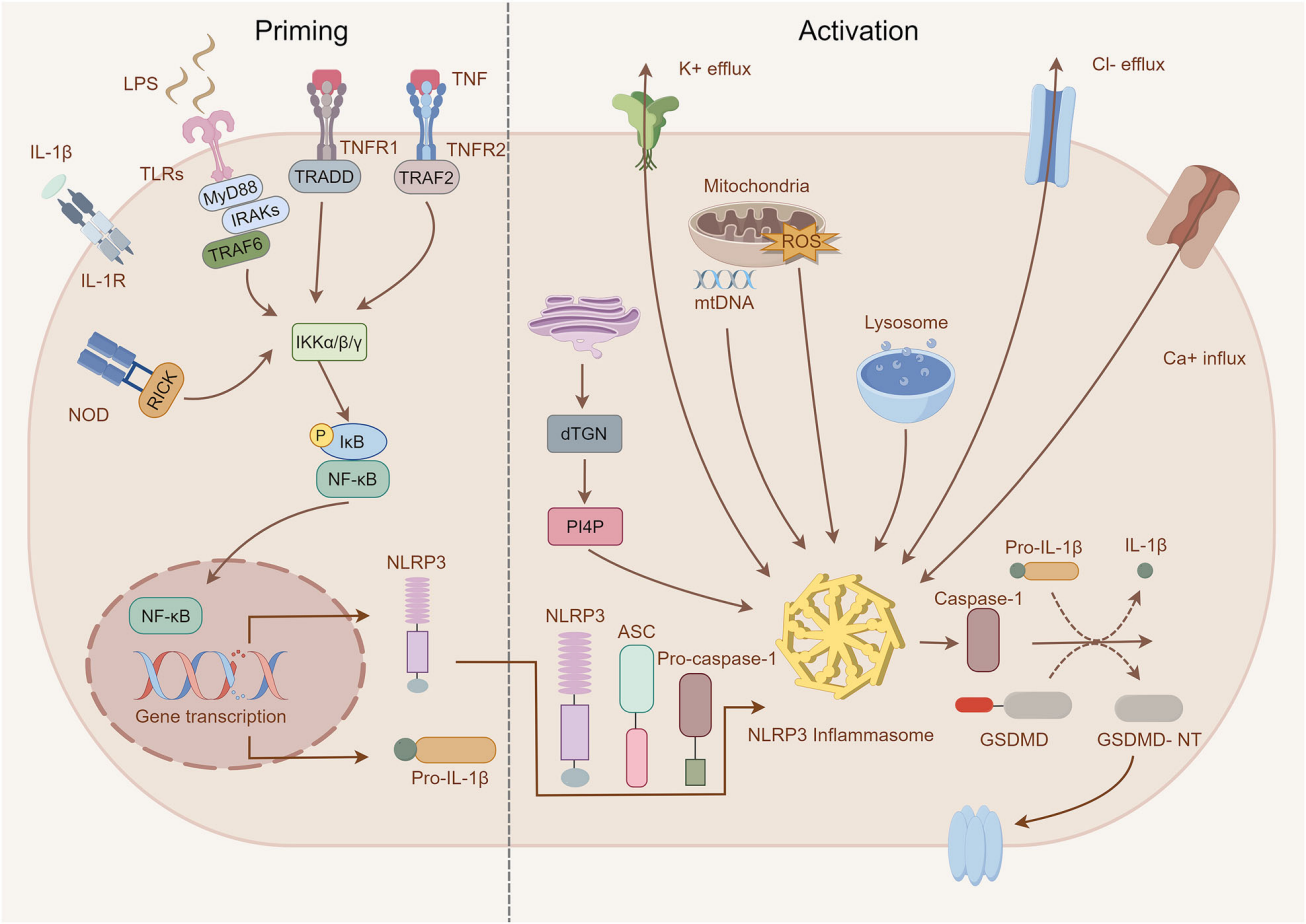
Mechanisms of canonical NLRP3 inflammasome activation. Canonical NLRP3 inflammasome activation generally involves two steps: the priming (left) and the activation (right). Induction of the priming step by LPS or endogenous cytokines such as TNF and IL-1β through binding of membrane-bound pattern recognition receptors, leading to activation of the NF-κB pathway to upregulate pro-IL-1β and NLRP3 expression. The activation process involves multiple molecular and cellular events, including efflux of K^+^ or Cl^−^, Ca2^+^ mobilization, lysosomes destruction, mtROS production, and mtDNA release, and trans-Golgi dispersion, promoting the assembly and activation of NLRP3 inflammasomes. Subsequently, activated Caspase-1 induces the maturation of inflammatory factors IL-1β and IL-18, and cleaves GSDMD, releasing its N-terminal domain to form membrane pores.

Canonical NLRP3 inflammasome activation is thought to require sequential priming and activation steps due to insufficient baseline NLRP3 expression (Fig. 1). In the priming process, PRRs such as nucleotide-binding oligomerization domain-containing protein 2 (NOD2), toll-like receptors (TLRs), TNF receptors (including TNFR1 and TNFR2), and cytokine receptors (IL-1R and TNFR) detect PAMPs and DAMPs. This recognition triggers the nuclear factor-κB (NF-κB) signaling pathway, which increases the transcription of NLRP3 and key inflammatory precursors, pro-IL-1β and pro-IL-18 [18]. However, recent evidence has shown that the priming step also licenses NLRP3 inflammasome assembly and activation. While the exact processes behind priming and licensing remain elusive, it is widely believed that the priming step is necessary to sufficiently induce functional NLRP3. This induction is achieved by regulating NLRP3 post-translational modifications (PTMs), such as ubiquitination, acetylation, phosphorylation, and SUMOylation. These modifications are crucial for the NLRP3 inflammasome to be completely assembled and activated [19].

#### The activation step

The priming process of inflammasome activation is essential for the subsequent activation phase, which is generally triggered by the recognition of NLRP3 agonists and involves inflammasome assembly and complete activation (Fig. 1). Compared to most PRRs, which typically exhibit specificity for limited PAMPs or DAMPs, NLRP3 demonstrates a broad responsiveness to diverse stimuli. In general, NLRP3 is triggered by microbial (e.g., bacterial, viral) infections, DAMP-mediated inflammation, and exposure to various environmental irritants. However, NLRP3 typically does not directly interact with these stimuli. Instead, these stimulating factors induce cellular stress responses, which are then sensed by NLRP3. It has been documented that NLRP3 can sense cellular stress through a variety of pathways, including the release of potassium (K^+^) and chloride ions (Cl^−^), the mobilization of calcium ions (Ca^2+^), destruction of lysosomes, the production of mitochondrial reactive oxygen species (mtROS), mtDNA release, and trans-Golgi dispersion. Additionally, NLRP3 can be activated through protein kinase R (PKR) and mitochondrial antiviral signaling protein (MAVS) signaling [20–23]. Although there is already a substantial amount of data describing the upstream signaling pathways contributing to NLRP3 inflammasome activation, these pathways frequently overlap and occasionally contradict one another.

### Non-canonical activation

**Fig. 2:**
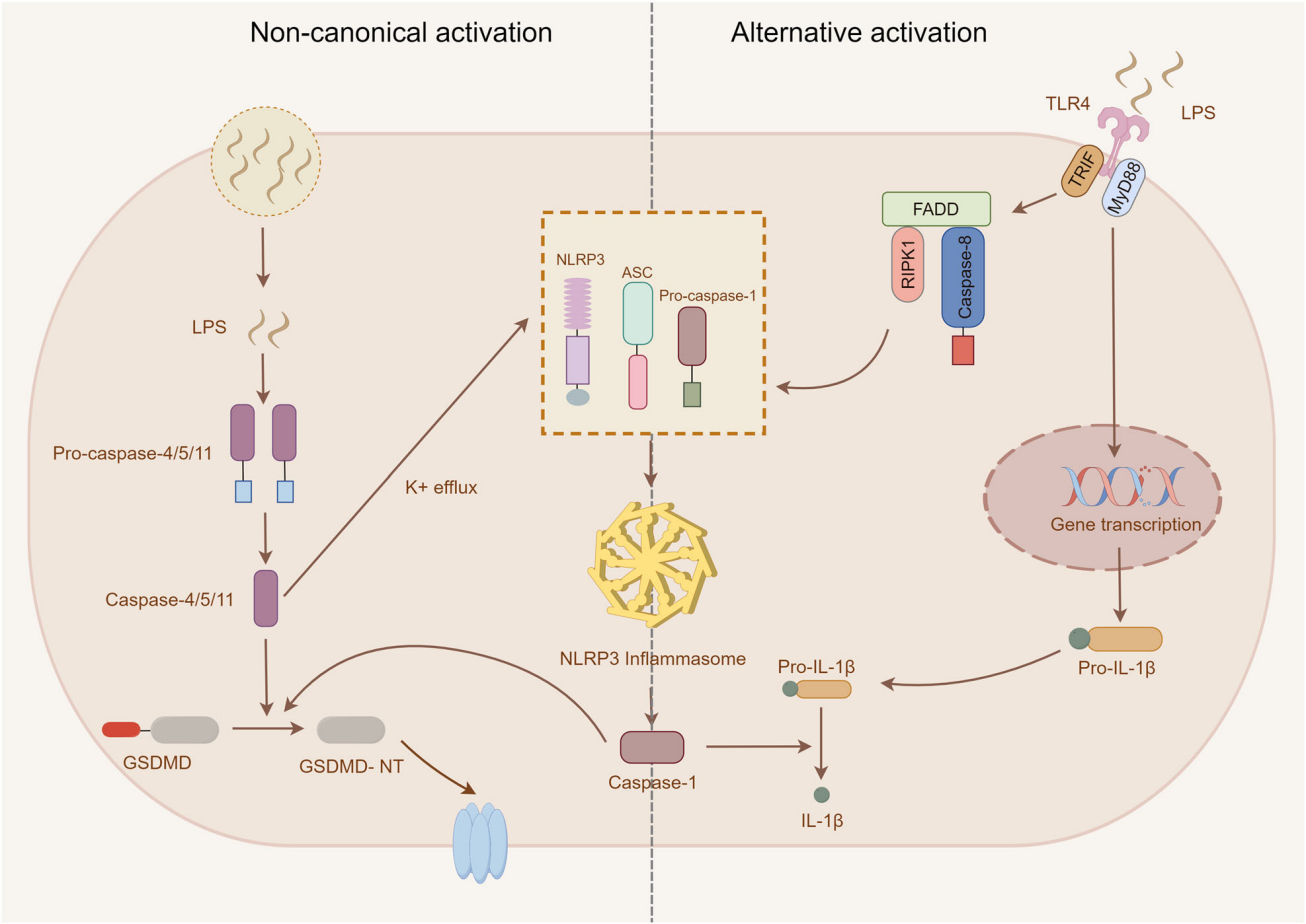
Mechanisms of non-canonical and alternative NLRP3 inflammasome activation. Non-canonical NLRP3 inflammasome activation(left) is induced by LPS released by gram-negative bacteria. After entering the cytoplasm, LPS directly induces human Caspase-4/5 or murine Caspase-11 to promote the cleavage of GSDMD. Moreover, this process is accompanied by K^+^ efflux-mediated activation of the canonical NLRP3 inflammasome. The alternative pathway of activation is induced by LPS in human monocytes, which directly activates NLRP3 inflammasome through the TLR4-mediated RIPK1-FADD-Caspase 8 signaling pathway without requiring K^+^ efflux or ASC speck formation.

Research indicates that Gram-negative bacteria are engulfed by phagocytic cells, which subsequently trigger the destruction of their bacterial walls, liberating lipopolysaccharide (LPS) and lipid A. LPS can specifically recognize and attach to the CARD domain of caspase-11 in mice or its human orthologs (caspase-4/5). This ligand-receptor interaction induces the oligomerization and self-cleavage of caspases, culminating in non-canonical NLRP3 inflammasome activation (Fig. 2) [24, 25]. Unlike canonical pathways, this activation mechanism bypasses the priming step due to constitutive expression of caspase-4 in human cells. Activated caspase-4/5/11 execute pyroptotic cell death via GSDMD cleavage, generating N-terminal fragments that perforate plasma membranes. Meanwhile, ATP release during this process mediates potassium efflux, which synergistically promotes canonical NLRP3 inflammasome signaling [26, 27]. While LPS remains the predominant activator, some activators from other sources have also been identified. For example, oxidized phospholipid 1-palmitoyl-2-arachidonoyl-sn-glycero-3-phosphorylcholine (oxPAPC) can interact with unique regions of murine caspase-11, distinct from those that bind to LPS, thereby activating non-canonical NLRP3 inflammasomes [28].

### Alternative activation

Unlike the two previously described activation pathways, LPS stimulation in human monocytes can trigger a distinct NLRP3 inflammasome activation pattern without requiring a second activation signal. In this process, there is no need for K^+^ efflux, which contrasts with the ATP-P2X7-dependent pathway, pore-forming toxins, and some particulate matter-induced NLRP3 inflammasome activation [29]. Mechanistically, this pathway activates NLRP3 inflammasomes through the TLR4-TRIF-FADD-caspase-8 signaling axis, independent of ASC speck formation and pyroptosome assembly [29]. These distinguishing features have led to its classification as the alternative (or one-step) NLRP3 inflammasome pathway (Fig. 2). In addition, in vitro models using murine dendritic cells exposed to LPS for an extended period demonstrated the capability to elicit NLRP3-dependent IL-1β secretion without relying on ATP-P2X7 signaling cascades [30].

## NLRP3 inflammasome in human diseases

Given that NLRP3 is an important protein involved in cell death and drives the pathophysiological mechanisms of many diseases, it has attracted considerable attention recently. Here, we discuss the impact of NLRP3 on autoimmune diseases, metabolic disorders, neurological conditions, fibrosis, and tumors (Table 1).

**Table 1:** Role of NLRP3 inflammasome in different diseases.

Diseases	Disease and pathologic feature	Role of NLRP3 inflammasome	Ref
Autoimmune diseases			
Systemic lupus erythematosus (SLE)	Systemic inflammation and dysfunction of multiple organs	Promoting the activation of B cells	[38]
Rheumatoid arthritis (RA)	Joint pain, swelling and bone destruction	Promoting the differentiation of proinflammatory T cells and inducing the activation of adaptive immune responses	[41]
Systemic sclerosis (SSc)	Immune dysregulation, endothelial dysfunction and progressive fibrosis	Regulating the activation of macrophages, B cells, Th cells, fibroblasts, and endothelial cells	[45]
Metabolic diseases			
Obesity	Chronic low-grade systemic inflammation	Reducing glucose uptake in insulin-target tissues (such as muscle and adipose tissue)	[51–53]
Type 2 diabetes mellitus (T2DM)	Hyperglycemia, hyperlipidemia and insulin resistance (IR)	Reducing glucose uptake in insulin-target tissues (such as muscle and adipose tissue)	[51–53]
Atherosclerosis	Atherosclerotic plaque formation	Mediating monocyte/macrophage aggregation and the proliferation of vascular endothelial cells	[56, 57]
Neurodegenerative diseases			
Alzheimer’s disease (AD)	β-amyloid plaque deposition, neurofibrillary tangles (NFTs) formation and chronic neuroinflammation	Regulating the formation of β-amyloid protein and the pathological changes of tau protein, and inducing inflammatory response	[62, 63]
Parkinson’s disease (PD)	Loss of dopaminergic neurons in the substantia nigra and α-synuclein-containing Lewy bodies	Mediating neuroinflammation, dopamine neuron loss and neurodegeneration	[67]
Multiple sclerosis (MS)	Oligodendrocyte loss and axons demyelination	Promoting the migration of CD4+ T cells, macrophages, and dendritic cells to the central nervous system (CNS)	[68]
Fibrotic diseases			
Various organs fibrosis	Excess extracellular matrix (ECM) accumulation and damage of parenchymal cells	Accelerating the expression of fibrosis-related proteins and the deposition of extracellular matrix (ECM); promoting epithelial-mesenchymal transformation (EMT)	[83, 84]
Cancer			
	/	Complex and context-dependent	[88]

### Autoimmune diseases

The main effectors of NLRP3 inflammasome activation are IL-1β and IL-18, which mature through caspase-1 cleavage. These inflammatory factors have an essential function in regulating the body’s immune response by affecting different immune cells [31]. Autoimmune diseases are pathologically defined by the disruption of immune tolerance and excessive immune responses, leading to specific or multiple organ damage [32]. Systemic lupus erythematosus (SLE) is a widespread autoimmune disease that typically manifests as systemic inflammation and dysfunction of multiple organs [33]. Research has shown that, compared to other groups, SLE patients—particularly those with lupus nephritis (LN)—exhibit increased NLRP3 inflammasome activation because of significant gain-of-function mutations in the *NLRP3* gene [34]. Moreover, upregulation of NLRP3 and IL-1β expression has been consistently detected across diverse cell types in SLE patients, such as renal tubular epithelial cells, macrophages, and peripheral blood mononuclear cells (PBMCs) [34–36]. Several inflammatory factors related to NLRP3 inflammasome activation, especially TNF-α, are upregulated in SLE[37]. TNF-α is a molecule of great interest that mediates SLE development by stimulating B cells to produce antibodies[38]. Rheumatoid arthritis (RA) exemplifies an autoimmune disorder, although its exact pathogenesis has not yet been elucidated. Clinically, RA is distinguished by joint pain, swelling, and bone destruction [39]. Investigations determined that NLRP3-mediated IL-1β/18 release has an active effect on RA pathogenesis [40]. Through IL-1β/18, NLRP3 in the synovium facilitates the differentiation of proinflammatory T cells and stimulates adaptive immune responses, further aggravating RA [41]. Systemic sclerosis (SSc) is a disease of the connective tissue with autoimmune origins, featuring immune dysregulation, vascular endothelial impairment, and progressive fibrosis. It is caused by genetic susceptibility and environmental triggers, particularly occupational hazards, viral infections, and chemical agents [42]. Endothelial injury initiates a cascade that releases ROS and inflammatory factors, which help activate NLRP3 inflammasomes [43]. Compared to healthy individuals, SSc patients show elevated expression of NLRP3 and downstream cytokines in skin tissue [44]. NLRP3 inflammasomes may contribute to SSc pathogenesis by modulating the activation of macrophages, B cells, Th cells, fibroblasts, and endothelial cells [45].

### Metabolic disorders

The mutual communication between immunological and metabolic systems is crucial for maintaining metabolic equilibrium. Dysregulated NLRP3 inflammasome activation leads to an imbalance between these systems and the release of inflammatory factors that mediate metabolic disorders, such as obesity [46], type 2 diabetes mellitus (T2DM) [47], and atherosclerosis [48], through autocrine or paracrine mechanisms. Obesity, marked by chronic low-grade systemic inflammation due to the excessive accumulation of fat, is also a key pathological mechanism that induces insulin resistance (IR) and T2DM [49]. T2DM has become one of the fastest-growing metabolic diseases in the world and is distinguished by hyperglycemia, hyperlipidemia, and IR. In an inflammatory environment, ectopic fat accumulation in the muscles and liver leads to IR and abnormal elevations of glucose levels in the blood [50]. Several studies have revealed that, compared to healthy individuals, the NLRP3 expression and its activated products, IL-1β/18, are upregulated in the visceral and subcutaneous adipose tissue of obese individuals [51, 52]. Additionally, the reduced levels of NLRP3 and IL-1β are related to improved insulin sensitivity brought about by caloric restriction and weight loss [53]. NLRP3 activation mechanistically facilitates the secretion of downstream inflammatory factors, including IL-1β, leading to IR that ultimately impairs glucose uptake in insulin-targeted tissues, including muscle and adipose tissue. This reduction in glucose uptake participates in the progression of obesity and diabetes [17]. A recent investigation highlighted the link between the NLRP3 inflammasome and diabetic angiopathy. Experimental data reveal that significant NLRP3 hyperactivation occurs in aortic tissues of T2DM murine models, while the absence or inhibition of NLRP3 effectively mitigates vascular aging triggered by T2DM [54]. Additionally, atherosclerosis, a chronic inflammatory disease, contributes to most cardiovascular diseases. Massive immune cell infiltration occurs in the arterial wall, along with lipid deposition and hyperproliferation of vascular wall cells, which are the main reasons for the formation of atherosclerotic plaque [55]. In an earlier study, scientists found that mice with atherosclerosis induced by low-density lipoprotein receptor deficiency exhibited reduced atherosclerosis when there was a deficiency of NLRP3 inflammasome elements in their bone marrow cells [56]. The NLRP3 inflammasome-activated products IL-1β and IL-18 mediate monocyte/macrophage aggregation and vascular endothelial cell expansion, both of which are important factors in atherosclerosis pathogenesis [57].

### Neurodegenerative diseases

Increasing evidence indicates that NLRP3 is a pivotal pathogenic driver across multiple neurodegenerative diseases, with demonstrated involvement in Alzheimer’s disease (AD) [58], Parkinson’s disease (PD) [59], and MS [60]. AD is an incurable and progressively worsening neurodegenerative disorder distinguished by the formation of β-amyloid plaques, the aggregation of hyperphosphorylated tau in neurofibrillary tangles (NFT), and chronic neuroinflammation [61]. Investigations illustrated that the NLRP3 inflammasome activation is linked to β-amyloid formation and pathological changes in tau protein, supporting the amyloid cascade hypothesis in AD [62]. Furthermore, the NLRP3 inflammasome triggers caspase-1 stimulation and IL-1β maturation, leading to inflammatory events [63]. Patients with PD usually exhibit signs of tremor, rigidity, and bradykinesia. The main pathological features include the depletion of dopaminergic neurons in the substantia nigra and the presence of Lewy bodies containing α-synuclein [64]. NLRP3, a central driving factor in neurodegenerative diseases, primarily facilitates the pathological advancement of PD through the assembly and activation of inflammasomes in microglia, thereby mediating neuroinflammation [65]. Yan et al. discovered that when the NLRP3 inflammasome is triggered in mouse microglia, it exacerbates dyskinesia and the loss of dopaminergic neurons in the brain. This effect can be counteracted by Parkin’s role in degrading NLRP3 through ubiquitination [66]. In addition, another study found that the NLRP3 inflammasome is autonomously activated in dopamine neurons, leading to cell death, which is a key mechanism contributing to dopamine neuron loss and neurodegeneration in PD [67]. MS is a neurodegenerative condition mediated by an autoimmune response that typically affects the central nervous system (CNS), with patients presenting with cognitive and motor dysfunction. The mechanism is primarily induced by the immune response of CD4 + T cells against myelin antigens, leading to several pathological alterations, such as oligodendrocyte loss and axonal demyelination [60]. The NLRP3 inflammasome facilitates the migration of various immune cells, including CD4 + T cells, macrophages, and dendritic cells, into the CNS, and is considered a crucial nexus between innate and adaptive immunity during early MS pathogenesis [68]. Furthermore, IL-1β released from NLRP3-mediated cell death is a risk factor that promotes susceptibility to MS and its progression, closely related to T cell differentiation [69, 70].

### Fibrotic diseases

Fibrosis is a prevalent pathological consequence of chronic inflammation in several organs, marked by excessive deposition of extracellular matrix (ECM) and the injury or loss of parenchymal cells resulting from the inflammatory response [71]. The NLRP3 inflammasome, a polyprotein oligomer integral to numerous inflammation-associated illnesses, contributes to the fibrosis of multiple organs, such as the hepatic, renal, cardiac, and pulmonary tissues [72]. Generally, NLRP3 activation can accelerate the production and secretion of inflammatory mediators, trigger the activation of effector cells, and upregulate fibrotic protein expression, thereby exacerbating the deposition of ECM. Experimental evidence from murine hepatic fibrosis models demonstrates that NLRP3-mediated hepatic stellate cell activation promotes the development of liver fibrosis [73]. Additionally, Kaufmann et al. discovered that NLRP3 activation was associated with macrophage recruitment and early liver fibrosis [74]. It is worth noting that the NLRP3 inflammasome was significantly activated in the renal tissue of UUO mice and in those with streptozotocin-induced diabetes. Inhibition of NLRP3 activation significantly enhanced renal function and mitigated renal fibrosis [75, 76], which is related to its influence on inflammatory factor production and podocyte activity [77]. Similarly, NLRP3- knockout (NLRP3-KO) mice maintained on a high-fat diet exhibited significantly less cardiac fibrosis than controls, indicating that NLRP3 is an essential protein that promotes cardiac fibrosis [78]. Chen and colleagues recently revealed that NLRP3 inflammasome activation enhances the migration and activation of cardiac fibroblasts, thereby exacerbating heart damage and accelerating fibrosis [79]. Notably, the NLRP3 inflammasome can also be triggered by several environmental pollutants, including fine particles (PM2.5) and radiation, both of which are important factors in inducing pulmonary fibrosis [80–82]. Research illustrated that the NLRP3 inflammasome enhances epithelial-mesenchymal transformation (EMT) and induces lung inflammation and fibrosis by activating TGF-β1/Smad pathway [83, 84].

### Cancers

Cancer has become a major public health issue that jeopardizes human health worldwide, with its occurrence and development being diverse and complicated [85]. Many investigations have revealed that inflammation significantly contributes to cancer progression, and the NLRP3 inflammasome is considered to be involved in this process due to its induction of pro-inflammatory cytokines, such as IL-1β and IL-18, and its activation of critical inflammatory signaling cascades, including the NF-κB pathway [86]. Research reveals that there is significantly enhanced NLRP3 activity in multiple oncological tissues compared to healthy control tissues [87]. The NLRP3 inflammasome complex exhibits context-dependent duality in tumorigenesis, demonstrating both pro- and anti-tumorigenic effects contingent upon microenvironmental factors [88]. For instance, NLRP3 inflammasome-triggered pyroptosis enhances the effectiveness of anticancer agents in colorectal cancer, while decreased NLRP3 levels are linked to a poor prognosis in the disease [89]. Conversely, in breast cancer, IL-1β facilitates the invasion, migration, and EMT of breast carcinoma cells [90]. Emerging evidence highlights the oncogenic potential of NLRP3 inflammasome activation via pyroptotic pathways in breast carcinogenesis [91, 92]. Moreover, investigations have confirmed that elevated NLRP3 levels are among the critical factors contributing to the growth and metastasis of cancer cells in various malignancies, such as lung carcinoma [93]. Considering the context-dependent dual effects of the NLRP3 inflammasome in different cancers, further investigation is essential to explore its specific contributions to cancer progression.

## NLRP3 inflammasome: an important component of PANoptosis

**Fig. 3:**
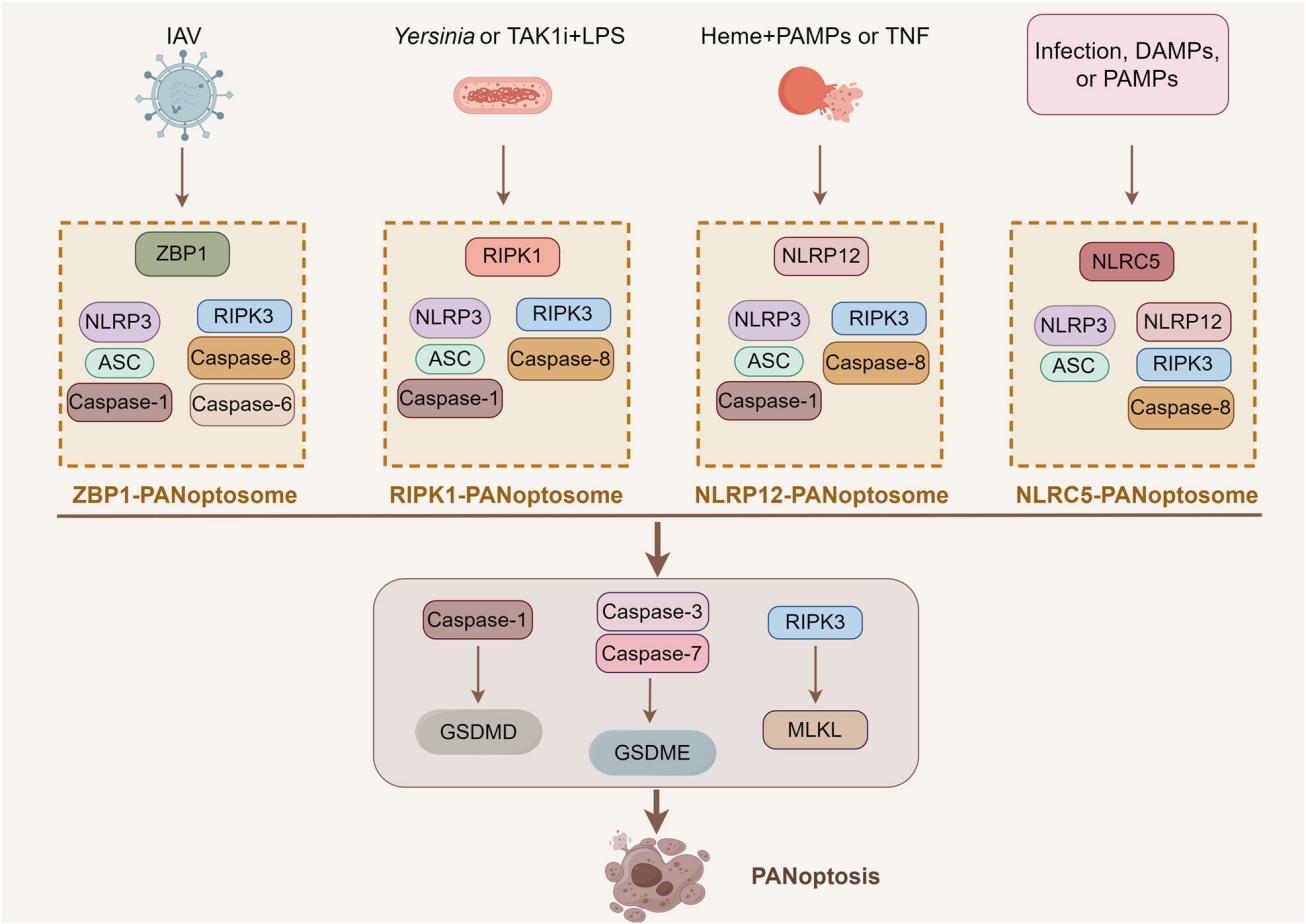
Molecular mechanism of NLRP3-dependent PANoptosis. When stimuli such as microbial infections or changes in cellular homeostasis are detected by intracellular sensor molecules, including ZBP1, AIM2, RIPK1, NLRP12, and NLRC5, certain cell death molecules are recruited to form a PANoptosome. NLRP3 is mainly involved in the formation of the ZBP1-PANoptosome (ZBP1, NLRP3, ASC, Caspase-1, RIPK3, Caspase8, and Caspase 6), the RIPK1-PANoptosome (RIPK1, NLRP3, ASC, Caspase 1, RIPK3, and Caspase8), the NLRP12-PANoptosome (NLRP12, NLRP3, ASC, Caspase 1, RIPK3, and Caspase8) and the NLRC5-PANoptosome (NLRC5, NLRP3, ASC, NLRP12, RIPK3, and Caspase8). These PANoptosomes further induce downstream Caspase 1 activation-mediated GSDMD cleavage, Caspase 3/7 activation-mediated GSDME cleavage, and RIPK3-mediated MLKL phosphorylation, promoting membrane pore formation and PANoptosis.

The term “PANoptosis” was originally proposed and defined in 2019 by a research team led by Professor Kanneganti [94]. Their research discovered a unique protein complex that can trigger a programmed cell death pattern, concurrently exhibiting features of pyroptosis, apoptosis, and necrosis [94]. This complex was later named the PANoptosome [95]. Prior to the introduction of the concept of PANoptosis, considerable evidence indicated significant crosstalk among the key regulatory molecules of certain cell death pathways [96–98]. The discovery of PANoptosis provides critical mechanistic insights into the crosstalk between various cell death processes. Previous research on the NLRP3 inflammasome has largely focused on a single form of cell death, for instance, pyroptosis. Nevertheless, recent investigations have identified the NLRP3 inflammasome as a key element in several PANoptosome complexes that induce PANoptosis (Fig. 3). PANoptosis, a pivotal effector mechanism in innate immunity, can be triggered through multiple pathophysiological stimuli, including microbial pathogen invasion, tissue damage, inflammatory signaling cascades, and oncogenic transformation [99]. Extensive research has identified the involvement of PANoptosis in infection pathology, autoimmune disorders, inflammatory diseases, and the development of cancer [13].

PANoptosis is mediated by specialized PANoptosome complexes that drive divergent phenotypic outcomes through distinct downstream molecular cascades. Currently, various PANoptosome complexes have been discovered, including Z-DNA-binding protein 1 (ZBP1), AIM2, NLR family pyrin domain-containing 12 (NLRP12), receptor-interacting serine/threonine-protein kinase 1 (RIPK1), and the recently characterized NLR family Caspase recruitment domain-containing 5 (NLRC5)- PANoptosomes [100, 101]. Notably, NLRP3 serves as a core regulatory component critical for the formation and activation of the ZBP1-, RIPK1-, NLRP12-, and NLRC5-PANoptosome. The ZBP1-PANoptosome, comprising ZBP1, NLRP3, ASC, Caspase-1, RIPK3, RIPK1, and Caspase-8, which were first identified in the context of influenza A virus (IAV) infection [102]. Mechanistically, ZBP1 acts as a sensor molecule that specifically detects viral RNA through its Zα2 domain. It subsequently recruits the NLRP3 inflammasome, inducing caspase-1-mediated pyroptosis and facilitating the liberation of inflammatory cytokines. Furthermore, the ZBP1-NLRP3 inflammasome can also interact with RIPKs and caspase-8 to assemble the ZBP1-PANoptosome. This process culminates in the sequential activation of caspase-3/7 and mixed lineage kinase domain-like pseudokinase (MLKL), which triggers apoptosis, necrosis, and PANoptosis [103, 104]. Additionally, recent reports have found that caspase-6 promotes the binding of ZBP1 to RIPK3, facilitating the subsequent assembly of the ZBP1-PANoptosome [105, 106]. Therefore, NLRP3 inflammasome regulates PANoptosis via two primary mechanisms: first, it directly induces pyroptosis and facilitates the maturation of inflammatory factors; second, it participates in PANoptosome assembly to execute PANoptosis processes. Similarly, the NLRP3 inflammasome contributes to the generation of the RIPK1-PANoptosome, which forms protein complexes with RIPK1, RIPK3, and Caspase-8[107]. Transforming growth factor-β (TGF-β)-activated kinase 1 (TAK1) is a crucial kinase that plays a vital role in preserving NLRP3 inflammasome inactivity and cellular homeostasis [108]. The RIPK1-PANoptosome complex is typically assembled in the absence or inhibition of TAK1 (TAK1i) and during Yersinia infection, while also requiring stimulation with LPS [109]. The NLRP12-PANoptosome complex, produced by heme combined with PAMPs or TNF stimulation, was found to contain NLRP12, NLRP3, ASC, Caspase-1, RIPK3, and Caspase-8, although the complex can still form in the absence of NLRP3 [110]. NLRC5 is an enigmatic NLR sensor responding to bacterial infections, DAMPs, and PAMPs, and is involved in regulating NLRP3 inflammasome activation. Recently, Kanneganti et al. identified that NLRC5 associates with NLRP12, NLRP3, Caspase-8, and other molecules involved in cell death after recognizing various stimuli, forming the NLRC5-PANoptosome to trigger PANoptosis [101]. Their research confirms the pivotal involvement of NLRC5-mediated cell death in causing tissue damage and inflammation [101]. It is particularly noteworthy that there may be other unidentified sensor-specific PANoptosomes that form under certain external stimuli or homeostasis disorders. Remarkably, although NLRP3 is acknowledged as the most canonical inflammasome sensor in the NLR family, its roles and functions in the formation of PANoptosomes still require further exploration.

## Targeting strategies for NLRP3

The undeniable link between NLRP3 inflammasome activation, cell death, and human disease makes NLRP3 inhibitors an important focus for future research. The potential of wide-ranging therapeutic applications for small-molecule NLRP3 inhibitors that are both selective and potent has prompted their development and discovery (Table 2).

**Table 2:** Compounds that target NLRP3.

Compound name	Structure	Clinical development stage	Indications	Ref
CRID3 (MCC950, CP-456773)		Phase II	Rheumatoid arthritis (RA)	[113]
ZYIL1		Phase II, NCT05186051	Cryopyrin-associated periodic syndrome (CAPS)	[115]
DFV890		Phase II, NCT04868968 Phase II, NCT04886258	Familial cold autoinflammatory syndrome (FCAS) Knee osteoarthritis	[118]
Selnoflast		Phase I, NCT04086602	Cryopyrin-associated periodic syndrome (CAPS)	[119]
Dapansutrile (OLT1177)		Phase II, NCT01768975 Phase II, EudraCT number 2016-000943-14 Phase II, NCT03595371 Phase I / II trial, NCT04971499	Early-stage osteoarthritis Acute gout Schnitzler syndrome PD-1-resistant advanced melanoma	[87, 124]
CY-09		NA	NA	[128]
JC-171		NA	NA	[129]
Colchicine		NA	NA	[130]
Glabridin		NA	NA	[131]
Oridonin		NA	NA	[132]

MCC950 is currently the most widely studied NLRP3 inhibitor [111]. It was originally named CP-456,773 (CRID3) and was discovered through phenotypic screening of monocytes or macrophages that produce IL-1β [112]. Due to its specificity as an inhibitor of NLRP3 inflammasome activation, rather than targeting other inflammasomes, MCC950 is widely used in animal models of inflammatory disorders involving the NLRP3 inflammasome [113]. Although MCC950 demonstrated excellent targeting, the phase II clinical trial for RA treatment was terminated due to apparent liver toxicity [113]. Regardless, the discovery of MCC950 provides inspiration and momentum for improved NLRP3 second-generation inhibitors, some of which are already in clinical trials. ZYIL1, an analog of MCC950, has been shown to be safe and effective as an NLRP3 small molecule inhibitor in phase I clinical trials [114]. Its phase II clinical trial in CAPS patients has recently been completed (NCT05186051), and the published outcomes illustrate that ZYIL1 administration greatly reduced the levels of inflammatory markers in these patients and improved their general health status [115]. DFV890, a recently developed NLRP3 antagonist, has demonstrated favorable tolerability profiles in clinical studies. Both single- and multiple-ascending dose trials in healthy volunteers revealed no safety concerns [116]. Although the compound failed to demonstrate statistically significant clinical improvement compared to standard care in a phase II trial for COVID-19-related pneumonia [117], a recent investigation by Shen et al. revealed its superior pharmacokinetic properties relative to MCC950 and demonstrated effective therapeutic efficacy in murine models of acute gout [118]. Currently, several phase II clinical trials are being conducted to evaluate the therapeutic efficacy of this inhibitor across multiple indications, including familial cold autoinflammatory syndrome (FCAS) (NCT04868968) and knee osteoarthritis (NCT04886258). Selnoflast (a CRID3 derivative), developed by Roche Pharmaceuticals, has successfully completed phase I clinical trials involving both healthy volunteers and patients with CAPS (NCT04086602) [119]. Selnoflast primarily inhibits the ATP/ADP exchange and ATPase activity of the NLRP3 inflammasome by attaching to its ATP-binding Walker region or adjacent areas. As a second-generation derivative of CRID3, Selnoflast features a piperidine moiety that replaces the isopropyl furan moiety in CRID3’s structure, thereby exhibiting enhanced pharmacological potency and improved functional properties[120]. Based on its good safety characteristics and preliminary efficacy data, this compound is poised for further development in treating systemic inflammatory disorders, including ulcerative colitis [121]. Dapansutrile (OLT1177) is an orally bioavailable NLRP3 inflammasome inhibitor that has been evaluated for safety in healthy subjects and has been found to be well tolerated [122]. It primarily inhibits the ATP-induced conformational changes of NLRP3 by specifically binding to the ATP-binding site in the NACHT domain, thereby preventing NLRP3 from interacting with ASC. This effect directly intervenes in the crucial initial step of inflammasome assembly, preventing the recruitment of ASC and the aggregation of pro-caspase-1 [123]. Phase II clinical trials investigating early-stage osteoarthritis (NCT01768975) and acute gout (EudraCT number 2016-000943-14) have revealed preliminary therapeutic efficacy [124]. Furthermore, an ongoing phase II clinical study is evaluating its therapeutic potential in patients with Schnitzler syndrome (NCT03595371). Although clinical investigations of single NLRP3 inhibitors in cancer therapy remain limited, a phase I/ II trial (NCT04971499) is currently underway to evaluate Dapansutrile combination with Pembrolizumab in patients with PD-1-resistant advanced melanoma[87]. Moreover, the latest research shows that in vitro and in vivo models of breast cancer demonstrate a better therapeutic effect with the combination of Dapansutrile compared to single-agent anti-PD-1 treatment [125]. Therefore, the combination therapy of NLRP3 inhibitors is anticipated to become a potential direction for future cancer treatments, including breast cancer.

Notably, before the identification of inflammasomes, the antidiabetic agent glyburide had already been shown to effectively suppress IL-1β release [126]. Subsequent studies have demonstrated that this drug selectively blocks NLRP3 inflammasome-mediated IL-1β release without influencing the activation of other inflammasomes [127]. Although several second-generation glyburide-derived analogs, including CY-09 and JC-171, have been developed, they all currently remain in the preclinical development phase [128, 129]. Furthermore, several natural compounds have been determined as inhibitors of NLRP3 inflammasome activation. These include colchicine (derived from *Colchicum autumnale*) [130], glabridin (isolated from *licorice*) [131], and oridonin (the primary bioactive constituent of the Chinese herbal medicine *Rabdosia rubescens*) [132], all of which have demonstrated anti-inflammatory effects via targeted suppression of the NLRP3 inflammasome [133].

## Summary and future perspectives

Since the discovery of the NLRP3 inflammasome as a key component of the innate immune system, there has been a rapid expansion in the understanding of its activation regulation, signal transduction pathways, and role in various diseases. These findings lay the foundation for further insights into inflammasome mechanisms in autoimmune, metabolic, neuropathic, inflammatory, and oncological conditions, and they offer potential directions for the development of future therapeutic strategies.

Recently, scientists have discovered a novel protein complex termed the PANoptosome, which mediates a distinct type of cell death called PANoptosis. This discovery explains the complex interrelationships among the three main PCD pathways—pyroptosis, apoptosis, and necrosis—that exist in various disease models. Substantial experimental evidence has established PANoptosis as a critical mediator in diverse pathological processes, including viral and bacterial infections, inflammatory diseases, autoimmune disorders, oncogenesis, and multi-organ dysfunction. While the precise mechanistic pathways are not yet fully characterized, emerging insights suggest potential pathogenic overlap with NLRP3-associated disorders [134]. For example, TNF-α, a key inflammatory factor that induces NLRP12-PANoptosome activation, has been reported to be significantly elevated in SLE patients, thereby accelerating the progression of the disease [37]. Sun et al. also illustrated that PANoptosis is closely related to the imbalance of immune homeostasis in SLE [135]. In the context of metabolic diseases, multiple inflammatory mediators and DAMPs induce PANoptosis in cells across diverse metabolic tissues, particularly in the liver and pancreas, thereby contributing to the pathogenesis of IR [136, 137]. Moreover, the regulatory impact of PANoptosis in the tumor microenvironment has been gradually explored, and it is believed to hold significant promise for future tumor therapies [138]. It would be interesting to further explore NLRP3’s specific function in the generation of the PANoptosome complex and its involvement in the regulation of PANoptosis in different diseases.

With the maturation of inflammasome studies, significant advancements have been made in comprehending NLRP3 regulation. At the same time, researchers are working to develop effective NLRP3 inhibitors, some of which are already being used in clinical studies for various diseases. Although small-molecule chemical inhibitors and natural compounds targeting NLRP3 presented considerable therapeutic efficacy and ease of administration, their safety profiles remain a critical challenge that requires urgent resolution. The potential toxicity observed in clinical trials of candidate drugs like MCC950 highlights the importance of safety evaluation during drug development. The primary mission of future scientists and biopharmaceutical companies is to design safer and more efficient NLRP3 inhibitors. Consequently, advancing our understanding of NLRP3 activation mechanisms, its contributions to cell death pathways, and its implications across various disease contexts remains imperative.
